# Twin-Screw Melt Granulation for Oral Solid Pharmaceutical Products

**DOI:** 10.3390/pharmaceutics13050665

**Published:** 2021-05-06

**Authors:** Seth P. Forster, Erin Dippold, Tiffany Chiang

**Affiliations:** Pharmaceutical Commercialization Technology, MMD, Merck & Co., Inc., Kenilworth, NJ 07033, USA; erin.dippold@merck.com (E.D.); tiffany.chiang@merck.com (T.C.)

**Keywords:** continuous processing, melt granulation, twin-screw granulation

## Abstract

This article highlights the advantages of pharmaceutical continuous melt granulation by twin-screw extrusion. The different melt granulation process options and excipients are described and compared, and a case is made for expanded use of twin-screw melt granulation since it is a flexible and continuous process. Methods for binder selection are profiled with a focus on rheology and physical stability impacts. For twin-screw melt granulation, the mechanism of granulation and process impact on granule properties are described. Pharmaceutical applications of melt granulation ranging from immediate release of soluble and insoluble APIs, taste-masking, and sustained release formulation are reviewed, demonstrating the range of possibilities afforded by twin-screw melt granulation.

## 1. Introduction

Granulation is often required to ease handling of fine, poorly compressible, poor flowing, low bulk density materials. Pharmaceutical products tend to be made using either dry granulation by powder compression or wet granulation using a binder, typically a polymer, in solution that is dispersed among other ingredients and then dried to remove the solvent and leave polymer bridges that hold the granule together. Melt granulation is analogous to wet granulation though the binder, a polymer or lipid, is heated above a softening or melting point so it can form bridges amongst the dry particles that solidify to form an agglomerate.

Melt granulation has been used in many industrial applications, including polymer, chemical [1], metal, glass [2], fertilizer [3] and food production processes. It has been used as a pharmaceutical process for some time with the use of powdered or molten wax in a low shear mixer to achieve sustained release [4]. Waxes and lipids like paraffin [5], glyceryl monostearate [6], glycerol palmitostearate [5], glyceryl behenate, stearic acid [7,8], hydrogenated oils [9] and polymers like polyethylene glycols [5] have been granulated using high-shear granulators with heat from jacketed bowls or frictional heating.

The expansion of twin-screw extrusion in pharmaceutical applications, generally for formulation of amorphous solid dispersions, has caused a large increase in interest for continuous granulation since the equipment can also be used for melt granulation. Continuous twin-screw melt granulation (TSMG) has been successful with the same binders used in high-shear melt granulation, for example, lipids [10], PEGs [11], and hydroxypropyl cellulose [12,13]. These applications show the versatility of melt granulation for granulation of moisture sensitive APIs for immediate release, for improved dissolution, and for taste-masking and sustained release.

Particularly in pharmaceutical applications, melt granulation is an underexploited process that may reduce product costs and improve process efficiency relative to other granulation techniques. It requires no drying step to remove solvents or water. This results in a more efficient process and allows for the use of excipients that are otherwise challenging to incorporate. Relative to dry granulation by roller compaction, TSMG tends to produce a narrower range of granule sizes, improved flow and compaction strength [14]. Compared with wet granulation, melt granulation avoids hydrolytic API degradation by avoiding water contact. Granule strength depends on the binder used. Melt granulation with polymer binders can result in stronger granules than wet granules. Melt granulation has been shown to improve the binder distribution in the granules. In the case of extended-release metformin, melt granulation improved tabletability and API coverage compared to traditional dry and wet granulation approaches [13].

Melt granulation is not appropriate for thermally labile APIs with the current binder and process options. Melt granulation might also be complicated by drug miscibility in the molten excipients resulting in phase changes or instability during shelf life.

## 2. Melt Granulation Process Options, Particularly Twin-Screw Melt Granulation (TSMG)

Melt granulation uses many of the process trains used for wet granulation, such as a high shear bowl system with impeller and chopper or a fluid-bed granulator. These batch processes require the addition of sufficient heating of granulating fluid, heated transfer lines and nozzles, or using conductive or convective heating to melt the binder. Melt granulation can be tested in the laboratory using a “melt and pour” technique to add the binder into the rest of the formulation using a mortar and pestle or a low shear planetary mixer, followed by sizing with a sieve or a mill.

High-shear melt granulation (HSMG) can be done by pre-heating the binder, feeding through heated lines and nozzles into heated bowls, or through frictional heating by the impeller and blade can distribute the binder in some cases. Then, a jacketed bowl can be cooled, or the batch discharged from the bowl to air cool. Granules formed by high-shear melt granulation tend to be larger, bimodal, and have a wide distribution of particle sizes compared to other melt granulation techniques.

Fluid bed granulation is another common wet granulation process that can be adapted to melt granulation [15,16]. Two main approaches have been demonstrated: either the binder is sprayed onto the substrate [17,18], in which case heated binder preparation, lines, and nozzles are required. The binder has to be warm enough and sprayed quickly enough to coat granules before cooling. Alternatively, the binder is blended into the batch and then gently heated to produce granules [19,20]. Comparing the same formulation, fluid-bed melt granulation (FBMG) produced larger, less dense, and rougher granules compared with HSMG [16]. Fluid bed melt granulation has even been used to make hollow granules [21]. A variation of FBMG, tumbling melt granulation, uses a rotary fluid bed with a spherical seed pellet as a starting material [22].

A variation on melt granulation is spray congealing or spray cooling [23,24]. This process uses rotary atomizers in modified spray drying equipment [25] or custom rotary disk atomization [16] to produce highly spherical particles for direct use or as a high-quality substrate for coating. Usually, a higher proportion of molten binder is required for this process relative to other melt granulation approaches. Some examples of spray congealing applications are taste-masking clarithromycin [26], sustained release of verapamil hydrochloride [27], and solubilization of indomethacin [28]. At least one commercial product (ZMAX^®^) uses this technology in order to delay release of azithromycin in order to avoid gastrointestinal side effects [29,30,31].

Compared with these other process techniques, twin-screw melt granulation is of particular interest since it can easily be adapted to continuous operation. Compared with batch processes, continuous processes have smaller process footprints, higher throughput, improved batch size flexibility by allowing scaling by time, and improved in-process monitoring and control. Continuous process development is more rapid and requires less material to test process conditions. Continuous processes are expected to result in fewer batch rejections and lower cost of production [32,33].

TSMG has improved control of material addition, heat, and shear compared to other granulation methods. It can be designed to greatly reduce material residence time at elevated temperatures, enabling higher process temperatures without causing API or excipient degradation. To reduce the risk of thermal degradation, granulation can be achieved by frictional heating only, referred to as twin-screw dry granulation [34]. Shear between the screws’ mixing sections and overflight regions results in very efficient mixing so that lower binder levels are required to achieve the same tablet strength and hydrophilic matrix gelling for sustained release, reducing the overall tablet size [13]. Compared with high-shear melt granulation, twin-screw melt granulation produced elongated, more porous, larger granules [35] which form stronger tablets. [36,37,38,39,40,41] Modular screw and barrel design common among twin-screw extruders allows for flexibility to produce the target granule density and particle size.

## 3. Binders for Granulation

Formulation excipient selection for melt granulation can dictate many aspects of the resulting granule and final product. Melt granulation formulation is typically comprised of API and binder, with additional excipients added based on desired function and downstream processing.

During binder selection, a consideration of the API properties, especially its melting point, thermal stability, and phase behavior, is required. The API morphology and particle size distribution can impact the flow of materials into the process and the output granule morphology and size, which could, in turn, change the product performance.

Many of the binders that have been used for fluid-bed melt granulation or high-shear melt granulation can be used for TSMG as well. TSMG typically has dry binder addition into the extruder as a pre-blend or powder co-feed, but work has begun on melt injection and pre-plasticization of binder prior to granulation. [42] Binders typically fall into one of two categories, lipid-based and polymer-based. Binder selection influences the final target performance of the drug, be it immediate release, modified release, or taste-masking, and must be selected both for final product functionality and desired processability. Highly soluble binders and excipient fillers can aid in immediate release products while enteric polymer binders can assist in delayed release formulations. The granule can be designed to achieve desired properties through binder selection, binder concentration level, and processing conditions.

### 3.1. Binder Properties

Lipid and polymer-based binders offer different benefits and downsides, based on API properties and final target product attributes. Lipids offer low melting points and can provide hydrophobic environments to protect from moisture sensitivity and enhance bioavailability [43]. Waxy lipids and surfactant binders often create well flowing slippery granules with poor compressibility and mechanical strength generation. Polymer binders often produce dense granules that plastically deform under compression [44]. Hydrophilic-lipophilic balance (HLB) is a measure of the surface-active molecule polarity of fatty acids and can be used to predict behavior of the binder in oral formulations [45,46]. HLB can be used as a reference to select an appropriate binder for the final product function, for example, low HLB indicating a good candidate for sustained release due to the binder having low aqueous solubility. Table 1 lists the water solubility and potential applications of materials based on HLB value range.

Binder selection dictates the final granule attributes and can vary significantly dependent on the binder properties. Low melting point binders like polyethylene glycol (PEG) require less time to melt, therefore can form larger final granule size with less fines from a larger initial particle size [47]. Higher melting point thermoplastic binders like hydroxypropyl cellulose (HPC) require longer residence times in the barrel during TSMG. Use of a larger binder particle size results in more granulation fines when compared with smaller particle size binder. Often, use of higher molecular weight binders correlates with larger granule size [48,49]. This converse effect can be attributed to the mechanism of binder distribution within the extruder. Fine particle size and lower viscosity binders often evenly disperse, whereas large particle size and higher viscosity binders have less intragranular porosity [50]. See Table 2 for a description of binders that have been evaluated for melt granulation.

### 3.2. Binder Selection and Applicable Tools

Proper selection of binder for a melt granulated formulation is needed to ensure desired performance. Due to the nature of TSMG extruders using higher temperature and shear, processing of sensitive APIs can result in higher degradant levels. Miscibility introduces binder plasticization, inhibiting granule growth via nuclei sticking together [65].

Plasticizers can be intentionally added to reduce the polymer viscosity and glass transition temperature, lowering the processing temperature and potentially widening the spectrum of APIs that can be processed. The plasticizer may reduce the melting point of the API, however, and should be tested for this effect [59]. Intentional selection of miscible binders can be used for solubilization of API, dissolving the material into liquid phase with the binder for potentially modified recrystallization upon cooling [43]. Miscibility is a significant consideration when selecting a binder as it can either be leveraged or avoided depending on the specific functions required, for example, API-binder miscibility improving solubility or immiscibility preventing incompatibilities [48]. One way to avoid API-excipient miscibility is to process above the melt temperature of the binder but below the melt temperature of the API.

Prior to processing, miscibility can be tested using differential scanning calorimetry (DSC) and Fourier transform infrared spectroscopy (FTIR) to look for changes in melting point or peaks in the mixture when compared with the pure drug [49,59]. DSC can be used to select processing temperatures to avoid API melting [8,47,49,57,59,62,65,68,69,70]. Additionally, hot-stage polarized light microscopy can provide visualization of any crystalline dissolution in liquid binder by maintaining elevated temperatures on a drug-binder binary mixture [49]. Visible change in size and shape of API crystals can indicate miscibility between the drug and binder. Testing of binder miscibility prior to processing provides a prediction of material behavior and interaction at a reduced rate of material consumption. After processing, X-ray diffraction (XRD) can be used to compare crystalline phase of primary materials versus final melt granules to understand any amorphous conversion during processing [58]. Differences in characteristic peaks of the API or binder pre- and post- processing can indicate amorphous content or form change generated from granulation.

Aside from binder-API miscibility, the viscosity of the binder is also an important consideration as it drives the processing temperature range on the extruder. Rheometers are used to measure the viscosity of the binder as a function of temperature to properly select the binder and target processing temperature [47]. Melt rheometers provide further understanding of binder behavior and performance, providing target temperature for optimal binder processing [49]. Rheoscopes can be used to visually observe the behavior of binders and formulation systems as a function of temperature and shear, giving further insight into binder behavior, granulation mechanism, and potential miscibility [70,75]. Generally, reduced melt viscosity aids in granulation through increased binder distribution [13].

### 3.3. Binder Case Studies

Compatibility of Ondansetron HCl drug substance with assorted binders was assessed using DSC and FTIR. Stearic acid acted as a plasticizer, lowering the glass transition temperature of the pure drug, and had no change in IR peaks when mixed despite the depressed melting temperature. FTIR showed overlapping spectra between mixture and pure drug [59].

In another case, a significant shift in DSC endothermic peak was seen when Glipizide drug substance was mixed with PEG, which is attributed to the miscibility of the drug in molten PEG. Although the impurities were slightly higher than those of other formulations, the Glipizide/PEG mixture impurities were still within ICH standard limits [68]. This study detailed that drug-binder interaction does not indicate incompatibility.

Moteyne et al. (2016) intentionally investigated the impact of drug-binder miscibility on immediate release formulations using PEG and Soluplus as binders with model drugs metoprolol tartrate (MPT) and caffeine anhydrous (CAF). MPT was miscible in both binders and a plasticizing effect was seen, lowering the temperature of deformation. Multiple processing conditions resulted in significant impact on granule properties for miscible formulations, whereas immiscibility showed specific dependence on throughput as a determination of ease of binder distribution. This study detailed that miscibility may require additional consideration to processing conditions than immiscible formulations to generate target granule attributes [65].

An acetaminaphen formulation was prepared using TSMG with hydroxypropylcellulose (HPC) as the binder. A binder level of 10–15% *w*/*w* was used, with oversized granules observed at higher levels. API amorphization was observed shortly after granulation though the API later crystallized [76].

A separate study was performed on a thermally sensitive API to identify a suitable binder. HPC, PEG 8000, and Compritol were selected as binder options. HPC granules were the most compactible, but the API in PEG 8000 and Compritol granules degraded less. API degradation in the HPC granules was linked to the higher thermal and mechanical stresses with higher required process temperature [49] and further related to mechanical energy input and API particle attrition [60].

## 4. TSMG Critical Process Parameters and Output Granule Properties

Pharmaceutical TSMG is a variation of twin-screw extrusion and has many of the same critical process set-up or design factors and process control parameters as amorphous solid dispersion manufacturing. The overarching goal is to consistently produce uniform, well flowing, granules of a reasonable size for downstream capsule filling or tablet compaction.

### 4.1. Process Set-Up: Feeder, Screw Design, Barrel Design, Material Feed Location, Die Design

For consistent granules to be produced continuously, the materials must be fed into the extruder in a controlled way. Powders, either individual components or pre-blends, are usually fed gravimetrically using a loss-in-weight screw feeder. Cooling in the feed zone is usually necessary to avoid bridging or melt blockage. Some binders have such low melting points so they cannot be added directly as powders but must be melted and pumped or sprayed into the barrel through a feed port.

Focusing next on the extruder setup, most twin-screw extruders have modular screw elements that can be moved on the screw to different process zones. Conveying elements with open channels are designed to move material through the screw and apply limited shear and product contact, so the material is not granulated much. Paddle-shaped kneading elements are commonly used to blend polymers with additives or pigments and to produce pharmaceutical amorphous solid dispersions. However, when used for TSMG, this type of kneading element tends to introduce more shear and local heating than is typically desired. For TSMG, usually only a few paddles are used, placed in a zone downstream of initial powder heating. Distributive mixing elements designs that blend and apply less shear may improve melt granulation uniformity and control. Downstream of the mixing sections, the screw can be designed to break large granules and cool down the batch prior to discharge.

An optimized screw design, that is the location and selection of the screw elements, is critical to achieve the desired extent of granulation and to avoid too much shear which would lead to local melting, overgranulation, or even degradation. Too much shear or heating can also mean not enough granule breakage occurs to produce uniform granules. Especially with low melting point or low viscosity binders, the blend can be squeezed between the screw and barrel or between the screw and the barrel making undesirable large ‘flakes’. The geometry of the elements and clearance between the screw and barrel also change when scaling up to larger equipment.

Barrel design is not typically critical though the location of addition of a binder or secondary component as a powder or a melt may impact the extent of granulation–for example, adding a powder binder late in the process would reduce the time at temperature versus earlier on. The barrel should be long enough to complete each process step, including softening the binder, mixing, and, if necessary, melting and mixing the API into the binder.

Die design is also less critical than for HME since the granules flow freely out of the barrel. However, the hot granules should not be constrained or compressed at the exit. Sometimes the use of additional supports and guarding beyond the end of the barrel allow the granules to fall more freely out of the extruder and support the end of the screw shaft.

### 4.2. Process Parameters: Temperature, Screw Speed, Feed Rate, Discharge and Cooling

For TSMG, the material temperatures and degree of fill are critical. The process zone temperature is set low in the feeding zones to avoid bridging or melting on the hopper and then increase to a temperature around the melting or softening point of the binder and below the degradation point of the API or formulation. Though process temperatures can usually be well controlled, frictional or shear heating is inherent to the process and can result in unexpectedly high local temperature increases. Therefore, the combination of thermal input from the barrel and the mechanical energy from the screw is important, as in twin-screw extrusion. Process development studies are typically required to confirm the impact of these factors.

The feed rate into the extruder is usually maximized for process productivity while maintaining control of the granule properties. The degree of fill is set by the feed rate per unit screw rotation and the densification of the granules through the process. As more material is fed into the extruder at the same screw speed, the screws fill more, a smaller percentage of the batch is in contact with the barrel at any time meaning less thermal conduction. This results in less of the binder melting and finer granules. Reducing the feed rate at the same screw speed has the opposite effect, producing larger granules. Increasing the extruder screw speed at the same input feed rate results in a shorter residence time distribution and less granulation though it can also lead to more high shear zones with increased local heating and more granulation.

After exiting the extruder, the granules may need to be cooled before storage to avoid additional granulation or clumping. A conveyor with gentle open or forced-air cooling is sufficient at small-scale. Cooled conveyor or tunnels may be needed at larger scales to promote heat transfer. After cooling, the granules are usually milled to reduce the size or remove lumps in the batch, similar to wet granulation. The resulting milled granules can then be blended with extra-granular excipients or lubricants and then compressed or filled into capsules.

### 4.3. Impact of Process Parameters on Formulation Quality Attributes

Some APIs will degrade when heated. The material temperature is controlled primarily by the input feed rate, screw speed, zone temperatures. Aggressive, high-shear sections can also increase the temperature of the formulation above the zone’s set temperature due to wall friction and viscous dissipation. Particle friction against the barrel, attrition, and the addition of abrasive formulation components can also cause local heating. Often overall process optimization can help avoid API degradation, but not, thermal degradation can be managed by using lower melting point binders, pre-plasticizing the binder [42], modifying the screw design [60], or adding the API later in the process.

Granule properties are highly dependent on the binder type, formulation, and process parameters. As mentioned in Section 3, waxy lipid or surfactant binders can make slippery, soft granules that flow well but compress poorly since binder is not mechanically strong. Compressible or soluble fillers may be added to waxy granules to improve tablet strength or speed disintegration. Melt granules made with polymer binders tend to be dense and hard which produces hard, plastically deforming tablets. Process parameters, particularly product temperatures and time at those temperatures, will impact the size and density of the granules. Low temperatures for shorter times typically producecrumbling, soft granules while high temperatures or longer times can mean hard granules.

Granule particle size distribution is often a critical attribute for downstream applications. For immediate release products, finer particles improve tablet compression but can cause poor flow and coarse particles tend to reduce final tablet strength. Taste-masked granules should be small enough to avoid the feeling of grittiness, ideally less than 200–300 µm [77]. Sustained release particles with varied size will have different exposed surface areas, and, therefore, different dissolution rates. The material and process choices can be balanced to produce granules that are hollow or with high intragranular porosity, which have been shown to improve tablet compaction.

Particle size is controlled similarly by managing the exposure of the nucleated granule to heat and shear in the extruder in the mixing zones and is modified by breakage in downstream extruder zones. The granule nucleation mechanism depends on the relative particle size of the binder and the other components as well as the viscosity of the binder [37,78,79]. Finer sized or lower viscosity binders favor distribution of the binder on the other components. Larger or more viscous binders result in ‘immersion’ with the binder concentrated at the center and therefore less intragranular porosity [50]. Granules grow by binder coalescence and break when mechanical forces exceed the granule strength. In TSMG, growth and breakage are in competition as the particles move through the extruder. When particles are pushed together in the screw, sheared by the screw and pushed against the barrel walls that causes granule growth. This is balanced against granule breakage in the cooled region of the barrel downstream of the granulation zones in the extruder or during milling post-granulation.

The process impacts morphology and surface roughness of melt granules as well, from spherical to elongated to flattened ‘flakes’. Flakes can be avoided by limiting the number and length of mixing zones to avoid constriction between the screw and the barrel. Control of the shape and granule surface roughness can impact granule flow in downstream operations like encapsulation and tablet compression [47].

For a similar analysis of process impacts on granules for TSWG, refer to [80,81,82].

## 5. Applications of Twin-Screw Melt Granulation

### 5.1. Immediate Release

Immediate release (IR) dosage forms are the most common orally administered drug delivery products [83]. Examples of IR products made by melt granulation are described below, with process details to illustrate typical approaches.

Melt granulation was used to overcome the stability issues of a moisture sensitive compound intended for IR delivery. The use of a lipophilic binder showed an improvement to the stability of an immediate release moisture sensitive dipeptidyleptidase IV inhibitor [8]. The drug substance exists as a hemihydrate crystalline monohydrochloride salt (theoretical water content of 2.4%) which is slightly hygroscopic at ≤75% relative humidity (RH) under ambient room temperature (25 °C). The drug substance undergoes degradation in presence of water. Additionally, excipients that contain high or appreciable equilibrium moisture content (>5%) at 25 °C and 75% RH adversely impact the stability of the compound. Melt granules were prepared in a 10-L laboratory scale top-driven high shear mixer. The drug to binder ratio was shown to impact degradation. As the drug to binder ratio increases from 1:1 to 1:4 moisture degradation decreased. Melt granules observed by SEM showed ability for melt granules to produce a light coating of lipophilic binder protecting the particle from moisture while maintaining the high solubility and immediate release characteristics of the crystal. Tablets prepared by the melt granules exhibited low weight variation, good tabletability, and low tablet friability and a comparable immediate release dissolution rate to tablets manufactured via dry granulation technique. Finally, a long term stability study showed degradation below 2%, and minimal change in assay, dissolution release at 30 min, and moisture up to 4 months at 40 °C/75% RH when stored in induction sealed HDPE bottles.

The feasibility of TSMG was determined to maximize the drug loading of metformin in an immediate release fixed dose combination (FDC) tablet and to leverage benefits of a continuous processing technique [61]. Two grades of hydroxypropyl cellulose (Mw 1,150,000 g/moL and Mw 100,000 g/moL) were studied as binders in addition to PEG3350, however PEG3350 was deemed an unsuitable binder as it formed large agglomerated masses rather than granules. The ratio of Metformin hydrochloride (MET) to Sitagliptin Phosphate (SIT) was kept at a constant 850:63 to correspond with the marketed MET and SIT FDC product with the MET drug load up to 85% *w*/*w*. The binder, MET, and SIT were mixed using mortar and pestle (10 g batch size) then fed into a co-rotating 20:1 L:D (process screw length to diameter ratio) 10 mm twin screw extruder using conveying elements and no die. Processing temperature was 130 °C and screw speed was set to 10 rpm. Melt granules were combined with Avicel PH102 using a Turbula mixer prior to compression into caplets. Melt granules formed caplets that weighed 80 mg less than the than the marketed FDC equivalent. Dissolution of caplets containing HPC melt granules showed full release within 10 min thus proving melt granulation is feasible of developing high-dose immediate release solid dosage forms for fixed dosage combination products. A comparison of solvent, fluid bed, and aqueous granulation approaches for high drug loading of metformin HCl showed that twin screw melt granulation achieved the desired release profile with less binder [13].

High shear aqueous wet granulation, roller compaction, and twin screw melt granulation were assessed to identify which granulation technique could improve the physical properties of a BCS class II API compound demonstrating hygroscopicity, low bulk density, and poor powder flow [14]. Color change of the API compound was observed with the high shear wet granulation technique indicating possible physical transformation or moisture degradation therefore indicating wet granulation not suitable for this API compound. The highest drug to excipient ratio that could be achieved for roller compaction was 0.6:1 with formulation consisting of API compound, microcrystalline cellulose, hydroxypropyl cellulose, croscarmellose sodium, colloidal silicon dioxide, and magnesium stearate. Whereas melt granules manufactured using an 18 mm Leistriz twin screw extruder followed by impact milling generated granules with a API:polymer ratio of 4:1 for Compritol 888 ATO or Polyethylene glycol (PEG 4000) and a 1:1 ratio for granules with Kollidon VA64 or Eudragit EPO. However, tablets prepared from granules containing Eudragit EPO did not meet immediate release dissolution crtieria with only a 6% drug release at 45 min. Tablets manufactured with twin screw melt granules containing Compritol 888 ATO, PEG 4000, and Kollidon VA64 all demonstrated greater than 80% drug release within 30 min. Finally, with regard to the physical properties of the granules, the twin screw melt granules generated fewer fines and demonstrated improved flow characteristics compared to the roller compacted granules.

An example of an approved immediate release melt granulated product is AbbVie’s recently (2020) approved Oriahnn^®^ which is a copackage containing a fixed-dose combination capsule of 300 mg of elagolix and two hormones for morning administration and an elagolix capsule for evening administration [84]. The previously approved elagolix product Orilissa^®^ is available as 150 mg and 200 mg tablets. A patent assigned to AbbVie [85] describes a melt granulation process with the amorphous salt of elagolix sodium at a >50% drug loading incorporated in a small proportion of PEG 3350 with the addition of sodium carbonate as a pH modifier and anti-gelling agent. The granules are compressed into tablets that are then placed in capsules together with a tablet of the other APIs. Other melt granulation binders like PEG, HPC, TPGS, and poloxamer are also claimed as potential binders.

Even with the melt granulation applications described above, there are relatively few publications showcasing use of TSMG for immediate-release dosage forms despite the noted benefits of continuous processing compared to batch processing. Additional publications comparing the benefits of TSMG for immediate release dosage forms to traditional wet and dry granulation techniques, comparisons of in vitro to in vivo bioavailability, and evaluating additional binders is recommended to further promote the potential of twin-screw melt granulation as an alternative granulation technique in the pharmaceutical industry remains.

### 5.2. Solubilization

As pharmaceutical treatments are getting more complicated and advanced, new chemical entities are increasingly becoming less soluble and permeable. Biopharmaceutical Classification System (BCS) II and IV drug compounds are more common and more difficult to formulate. A common approach to increase the drug solubility and dissolution rate is to produce an amorphous solid dispersion by producing an single amorphous phase of the API in polymer either by rapidly evaporating solvent to isolate powder from solution using spray drying or melt-quenching using hot-melt extrusion (HME).

TSMG uses the same equipment as HME, but differs in the direct production of discrete, multiphase granules instead of a single-phase rod or flake that is milled to produce granules. TSMG also usually uses lower process temperatures and very low outlet pressures since there is no die to constrain flow at the discharge. TSMG uses less viscous polymers and lower temperatures than conventional HME. TSMG can be used to effectively disperse API into semisolid excipients, such as PEGs, poloxamers, and PEGylated lipids. The API is either maintained as crystalline particles, dissolved in the liquid phase with complete or partial recrystallization. An increase in dissolution rate can occur when the size of the crystals that form during cooling is reduced, or if a cocrystal or eutectic of the API with the binder forms.

Many published formulation studies use a benchtop preparation method with complete melting of the semisolid binder, dissolution of the API into the binder, then cooling, milling, sieving. This approach is simple and effective as a screening tool. Compared to this screening approach, TSMG is a more commercializable process, because it involves shorter contact between the binder and API and less time at temperatures. Moving to TSMG, control of the physical form may not translate directly. Therefore, the phase state and mechanism of solubility enhancement is important to understand through development.

Polyethylene oxide also known as polyethylene glycol (PEG) is a common excipient, available across a wide range of molecular weights. Solid grades with molecular weights of 1–10 kDa, that have a low melting point and high aqueous solubility, are used for melt granulation. Griseofulvin with PEG was an early application of solubility enhancement by melt granulation, with publication of the preparation in 1969 [86], initial FDA approval in 1975, and still marketed as “Gris-PEG^®^”. The product is described as ultra-micronized to differentiate from non-bioequivalent formulations of griseofulvin. More recent investigation of the physical form indicate that it is likely a cocrystal [87] and the molecular weight of the PEG impacts dissolution [88]. Indomethacin has also been dispersed in PEG 4000 via FBMG with apparent recrystallization after heating. This produces dissolution enhancement attributed to increased API surface area [89]. TSMG of ibuprofen and caffeine with PEG 3350 and 8000 at 100 °C barrel temperature avoided thermal degradation of either API and thermally induced polymorphic change of caffeine, attributed to the short residence time in the extruder. PEG can also be used as a binder during preparation of cocrystals with increased solubility to the orginial API [90].

For lipophilic insoluble APIs, surfactants may also improve dissolution rates. Poloxamers are a class of nonionic surfactants composed triblock copolymers of hydrophobic polypropylene oxide with hydrophilic polyethylene oxide invented in the 1970s. They are designed with different molecular weights of each domain. Solid grades poloxamer 188 and 407 are the most commonly used for oral pharmaceuticals and have low melting points suitable for melt granulation. Melt granules of Ibuprofen with poloxamer 188 produced by HSMG formed a single-phase eutectic as a result of processing and enhanced dissolution [69]. Similar results were observed with ibuprofen and poloxamer 407 [91,92] and etoricoxib with poloxamer 188 [93].

Gelucires are surfactants that form by PEG esters of fatty acids with grades identified by melting point/HLB. Of these, hydrophilic grades 50/13 (Stearoyl polyoxyl-32 glycerides) and 44/14 (Lauroyl polyoxyl-32 glycerides) are used most commonly for immediate release formulations [46]. These have been used, for example, to improve dissolution of carbamazepine [94], everolimus [95], gliclazide [96], olanzapine [97], and cefuroxime axetil [98] with granules made by the melt–stir–sieve process, for diazepam using HSMG [99], and meloxicam by fluid-bed coating onto beads [100]. For carbamazepine in particular, the rate-determining step in drug release was dissolution of Gelucire 44/14 and not ratio of drug: excipient. Additionally, it was observed that Gelucire 44/14 must be in particulate form rather than monolithic form to obtain an immediate release tablet formulation as compaction into tablets showed a significant decrease in drug release rate. Recently, a fully soluble surfactant Gelucire 48/16 (polyethylene glycol monostearate) was developed, with demonstrated solubility improvement when spray congealed with indomethacin [28].

Other solid surfactants with low melting points have been used for melt processing of poorly soluble compounds, including Vitamin E tocopherol polyethylene glycol succinate (TPGS) [101] and sucrose laurate [102].

Several publications compare these different excipients, focused on both physical stability and dissolution enhancement. Carvedilol was melt granulated with PEG 4000 and Poloxamer 188 with equivalent results though for both fluid-bed and high-shear granulation adding the binder in the initial charge (“melt in”) did not perform as well versus spraying onto the charge in the molten state (“spray on”). The API remained crystalline but wetted more effectively since the API surfaces were coated with the excipient [103]. HSMG of griseofulvin and PEG 3350 or Gelucire 44/14 with HPMC added with the intent of inhibiting API precipitation during dissolution showed increased dissolution rate and extent over API alone with apparent partial dissolution into PEG [104]. For praziquantel, Gelucire 50/13 improved dissolution more than poloxamer 188 or PEG 4000 for HSMG and ultrasonic spray congealing [105]. Investigational drug Lu-X was tested with Gelucire 50/13, PEG 3000, and poloxamer 188 using melt-in and spray-on during HSMG [106]. Carbamezine was twin-screw melt granulated with PEG 6000, poloxamer 407, as well as polyvinyl caprolactam-polyvinyl acetate-polyethylene glycol graft copolymer (Soluplus) [107].

### 5.3. Taste-Masking

Patient compliance is a profound public health challenge. One review estimated patients in developing countries are non-compliant an average of 50% of the time for a chronic treatment [108]. In many cases non-compliance leads to inadequate efficacy or disease mitigation and, particularly for infectious diseases, leads to resistance that is detrimental to the patient and public health [109].

**Figure 1:**
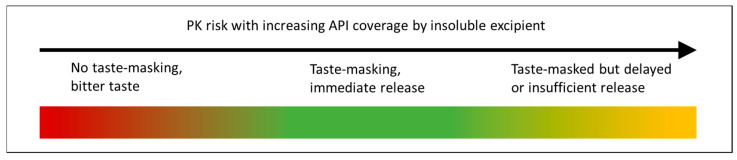
Schematic of balance between effective taste-masking and pharmacokinetic (PK) risk for dissolution barrier approaches.

Taste-masking an API can allow for a more patient-friendly orodispersible formulation, since, particularly for pediatric patients, tablets can be difficult for patients to swallow [110]. Regulatory authorities now expect development of pediatric formulations, including palatability assessments [111,112] since children have different preferences and needs as patients [113]. When developing a taste-masked dosage form, a bioequivalence (BE) strategy is typically preferred unless an improvement in onset time or efficacy is expected due to buccal or sublingual absorption or rapid disintegration. Referring to Figure 1, a BE approach requires an understanding of the trade-off between slowing the dissolution of the API in saliva and allowing for sufficient release in the gastrointestinal tract (GI), though for some drug products, sustained release and taste-masking may both be required. Taste-masking still be achieved while also improving the release of insoluble APIs [114].

Many taste-masking formulation technology options are available [115,116,117,118]. Among these, melt granulation is well suited to taste-mask APIs by matrix encapsulation. As the molten binder is heated, it tends to preferentially cover the outer surface which reduces the exposed surface API which in turn reduces drug release at the same drug loading level [12,37]. Lipids, hydrophilic matrix-forming polymers, and the amino-methacrylate copolymer Eudragit E are well suited to processing for this purpose. Of the excipient options shown in Table 2, those that do not dissolve quickly in water or melt at temperatures <40 °C are preferred for taste-masking.

Lipid excipients are versatile since they generally have low melting points, low viscosities as melts, and can be functionalized to achieve many different goals [43]. Many lipids have been used for drug delivery [119]. Lipids have been used to achieve taste-masking using varying processes [120], including formulation in a medicated chocolate [121]. Several examples of taste-masking formulations with lipid excipients are described below, most using processes other than TSMG, though continuous melt-granulation of these may be feasible and beneficial for the reasons described previously.

Saturated fatty acids such as stearic acid (C18, melting point of 70–72 °C) and palmitic acid (C16, melting point of 61–63 °C) were used for spray congealing with benzoic acid as a model API [122,123]. Zinnat^TM^ (cefuroxime axetil) oral suspension is comprised of API and stearic acid microspheres designed to avoid unpleasant taste by coating the API which delays release [124,125,126]. Similar formulations of this API have been made by melt-quenching [127].

Glyceryl monostearate (GMS), a commonly used food emulsifier and thickener, has been used for taste-masking, for example, with ondansetron hydrochloride [55] and satranidazole [128] using a melt-quench process followed by sieving, with clarithromycin in combination with a polymer using spray congealing [26], and with praziquantel via spray drying and hot melt extrusion followed by milling and sieving to less than 400 μm [129]. For praziquantel, taste-masking performance was most effective with hot melt extrusion. GMS allowed for a lower overall process temperature.

Glyceryl behenate (Compritol^®^ 888 ATO, Gattefossé) is a mixture of mono-, di and tribehenate (C22) of glycerin that has a melting range between 65 °C and 74 °C [45]. Glyceryl behenate has high batch-to-batch consistency and sharp crystalline melting and recrystallization temperature which assist with process control and consistent dissolution of resulting microparticles. This excipient has precedence of use for taste-masking and sustained release formulations, for example with azithromycin [31,130], diclofenac sodium [131,132] and tramadol hydrochloride [53]. Compritol^®^ 888 ATO has been extensively used in hot-melt coating applications, e.g., [133,134,135,136]; however, since it is insoluble it can delay release more than required for taste-masking and reduce bioperformance. It has a higher melting point and therefore higher granulthan is ideal for some APIs.

Another binder for taste-masking is glyceryl palmitostearate (Precirol^®^ ATO 5, Gattefossé), which has a lower melting point, between 50 °C and 60 °C, and exhibits rapid recrystallization upon cooling below 35 °C. Fluidized spray coating of Precirol onto acetaminaphen was patented [137], including claims for a wide range of APIs, and the process was optimized for taste-masking performance without compromising bioperformance [138]. A prolonged high-shear coating process that uses frictional heating to coat the API particles with Precirol is demonstrated and compared to fluid-bed coating with potassium chloride as an example API [139,140]. Micrographs of these particles show the vertices of the cubical crystals makes this material particularly difficult to coat, and the high-shear process covers the particles more effectively than fluid-bed coating, where the individual congealed spray particles can be seen instead of a uniform coating. Similar results were observed with acetaminaphen [141].

Polymeric excipients are also commonly used for taste-masking applications [117]. Use of these excipients depends on either delaying API release by using cellulosic excipients like insoluble ethylcellulose or hydrophilic gelling hydroxypropyl cellulose (HPC) or hypromellose (HPMC) or the use of polymers that are soluble at low pH of the stomach but not in the higher pH of saliva, also known as reverse enteric polymers, usually Eudragit^®^ E.

Cellulosic polymers were used to taste-mask caffeine citrate via hot-melt extrusion with milling to pelletize using ethylcellulose and soluble polymers as pore formers [142]. Caffeine citrate was also extruded with HPC and both with and without Eudragit^®^ E into filaments used to 3D print toroidal tablets [143]. Sildenafil citrate was taste-masked by extrusion with ethylcellulose, with the addition of crystalline calcium carbonate to adjust the local pH to reduce API solubility in saliva [144].

Eudragit^®^ E is cationic amino methacrylate copolymer that is soluble at acidic pH but not at neutral pH. It has frequently been used as a barrier coating for taste-masking, but this process requires multiple steps and a high-quality substrate for coating. Eudragit^®^ E is a good candidate for melt processing since it has a low glass transition temperature (about 50 °C) and is thermally stable. As a potentially simpler process, high-shear melt granulation of acetaminaphen with 10% *w/w* Eudragit^®^ E resulted in differential pH dependent release [145]. Twin-screw melt granulation was used to produce an amorphous solid dispersion of ibuprofen with Eudragit^®^ E increasing dissolution rate and avoiding API release at neutral pH, shown by in vitro dissolution and a human taste study [146]. Extrusion was used to produce taste-masked solid dispersions of Eudragit^®^ E with acetaminaphen [147], mefenamic acid [148], and efavirenz [149]. In vitro and in vivo taste assessments of mefenamic acid across drug loadings indicated that taste-masking performance was reduced at higher drug loadings, potentially due to the presence of crystalline API that would not be molecularly dispersed in the polymer [150]. A related approach, extrusion with milling to <500 µm, resulted in taste-masked extrudates of cationic APIs cetirizine hydrochloride and verapamil hydrochloride with anionic Eudragit^®^ L polymers [151].

Most of the formulation and process studies described in this section do not use melt granulation, but rather fluid-bed, high-shear, or hot-melt extrusion. Even so, the excipients are suitable for twin-screw melt granulation and could potentially be used for taste-masking APIs via TSMG. TSMG tends to produce APIs embedded in granules, however, and may not be suitable for highly aqueous soluble or extremely bitter compounds. In those cases, direct or post-granulation coating may be necessary.

### 5.4. Delayed and Sustained Release

Delayed release (DR) products are made to focus drug delivery on certain parts of the gut. DR formulations may employ enteric polymers to avoid release at the low pH of the stomach that may cause API degradation, reduced exposure due to pH-dependent solubility or they may be attempting to release the drug in a region of the gut that will improve or extend the exposure of the drug. Examples of formulation approaches for DR are enteric coated tablets, osmotic tablets, multiparticulates coated with coatings that dissolve at different pH’s, or amorphous solid dispersions with enteric polymers.

Eudragits are a class of polymethacrylates, among them several enteric grades of different pH-dependent solubilities (pKa’s from 5.5 to 7.0) that can be used to adjust drug release from the upper GI to the colon [152]. Several of these grades have been processed using HME [153,154]. They can be used for melt granulation, for example, posaconazole HSMG with Eudragit L100, with triethylcitrate as a plasticizer, and PEG 6000 as a binder [155]. These polymers could potentially be used with TSMG as well.

Ref. [156] Sustained release (SR) products spread the release of the drug along the gut and over time to extend the duration of therapy per unit dose. Frequently the goal of this formulation is to reduce dosing frequency from two or three times per day to once a day. SR formulation approaches include matrix tablets made with hydrophilic gelling polymers like hypromellose or poly(ethylene oxide), osmotic tablets, or multiparticulate systems containing water insoluble or gelling polymers or lipids.

Several lipidic melt granulating binders like diglycerides and fatty acids are also effective sustained release matrix forming excipients. Hydrophobic Gelucire grades 43/01, 39/01 and 33/01 have been used for delayed release with a range of processes and dosage forms [46]. HSMG has been used to produce pellets of phenylephrine hydrochloride as well as other model APIs with high levels of melt binders Compritol and Precirol to produce large pellets. [157] This process required careful temperature control to avoid rapid granule growth. TSMG has the potential to benefit this process since it allows for finer temperature control. Metoprolol succinate and Compritol 888 ATO were melt granulated using HSMG. This process resulted in an extended controlled release profile comparable with tablets made by direct compression or wet granulation with same Compritol levels. The MG tablets had acceptable friability and did not swell like the reference product [158]. Using TSMG, theophylline released over 24 h from matrix tablets with Compritol, Precirol, or Geleol^TM^ (mono and diglycerides) [52]. A double melt process dispersed granules of mesalazine and carnauba wax inside stearic acid pellets [156].

Rate-controlling polymers and lipids can also be effectively combined with non-melting sustained release excipients like controlled release grades of hypromellose or polyethylene oxide. The use of a molten binder avoids the challenge of drying these hydrophilic polymers after wet granulation. Some examples of this formulation approach are: zolpidem tartrate with HPMC K4M or PVP as a rate-controlling polymer and PEG 6000 as a meltable binder using the benchtop heat and mix method followed by sieving and compression [159], lovastatin granulated with HPMC K4M and PEG 6000 as a binder using HSMG [160], TSMG of metoprolol hydrochloride with stearic acid and PEO WSR N12K [72], and verapamil hydrochloride which was TSMG with either Compritol 888 or Precirol with HPMC K4M or PEO 1M [54]. Melt granules have been blended with another matrix forming excipient and tableted, for example, tenofovir and Gelucire melt granules were combined with HPMC or chitosan to make mucoadhesive tablets with a 7 day release for HIV prophylaxis [62].

Both DR and SR formulations are more challenging to develop and have increased regulatory requirements [161] over conventional immediate release products. Excipients and process can play a critical role in functionality. These formulations carry the risk of high exposure from APIs that dissolve in water or ethanol, known as dose dumping. Selecting ethanol insoluble excipients and coating the API by granulation can reduce this risk, as described for APIs generally [162] and with TSMG of tramadol hydrochloride with glyceryl behenate specifically [53]. Modified release formulations also carry the risk of low exposure if the API is insoluble at gastrointerestinal pH’s or if the targeted pH is not reached in vivo, for example, with products that are co-dosed with proton pump inhibitors which increase patients’ gastric pH.

DR or SR formulations produced by melt granulation use similar materials as described above but tend to focus on higher molecular weight lipids and polymers. Depending on the formulation, the granules may be delivered as granules, coated to further slow or delay release, or compressed to form matrix tablets. Compared with conventional approaches, melt granules tend to have more uniform distribution of the binder and matrix forming excipient and may be able to prolong release. This allows for a reduced rate-controlling polymer and therefore a higher drug loading and smaller tablet image size [12]. Plasticizers can further improve the API coverage by the binder due since they reduce the polymer viscosity and glass transition temperature as they dissolve into the polymer. In some cases, matrix encapsulation of the drug may be sufficient to avoid overcoating with a rate-controlling membrane, simplifying the process train.

### 5.5. Other Oral Formulation Applications

Effervescent tablets require very low moisture during processing and storage to avoid the acid-base reaction between the excipients. Melt granulation of effervescent formulations is attractive since it is a dry process. FBMG of an effervescent excipient with PEG 6000 [163] showed promise. The formulation could likely be made by TSMG. An extension of this idea uses embedded effervescent excipients to produce gas bubbles in a polymer matrix to make tablets float in the stomach for extended release [164].

## 6. Summary and Gaps in Understanding

Twin-screw melt granulation is a promising and versatile continuous process. It can be used in place of wet granulation to avoid the use of water, avoid the need for drying, and improve tabletability. Melt granulation is a simpler and more effective means to incorporate many functional lipid and polymer excipients. TSMG can also help realize the operational advantages of continuous processing.

While a wide selection of binders is available and described in these applications, the development of new binders, particularly that can be processed at relatively low temperatures, do not interfere with API dissolution, and are unlikely to be miscible with APIs and impact the physical form in the granule, would enable more robust processing of TSMG. Furthermore, improving the understanding of how to select binders and how to optimize the process around output granule properties would help support investment in this technique which in turn will increase understanding and TSMG availability for implementation. The impact of granule properties on downstream dosage form processing also requires additional research [58].

TSMG could be used for other applications that have not yet been tested or disclosed in the literature. Melt granules could also be a potential feedstock for emerging pharmaceutical thermal processing, like profile extrusion [165], injection molding [166], or cast molding [167]. Formulation for oral delivery of non-small molecule APIs, like peptides, antisense oligonucleotides, or proteins, has focused on lipid excipients as solubility and permeation enhancers [168,169], many of which could potentially be used for TSMG. Melt granules could be used for non-oral dosage forms like long-acting injections as microparticles [170] or implant rods [171].

Altogether, melt granulation is a process with unrealized potential and worthy of additional research and evaluation.

## Figures and Tables

**Table 1:** Application based on HLB, adapted from [46], Future J. Pharm. Sci. 2018.

HLB Range	Water Solubility	Application
1–3	Not dispersible in water	Sustained release
3–6	Poorly dispersible in water	Water in oil emulsifier
6–8	Coarse emulsion	Wetting agent
8–10	Stable emulsion	Oil in water emulsifier
10–13	Micro-/nano-emulsion	Surfactant
>13	Solution	Solubilizer

**Table 2:** Binders Used in TSMG.

Binder (Common Application)	Grade	HLB	Tg (°C)	Tm (°C)
Carnauba Wax (SR) ^1^	Carnauba Wax [23]	- ^2^	-	82–86 [23]
Castor oil, hydrogenated (IR) ^3^	Cutina^®^ HR [8]	-	-	85–87 [8]
Ethyl cellulose (SR)	Ethyl Cellulose 100cP [12]	-	133 [12]	-
	Aqualon™ 10 [51]	-	150–156 [51]	-
Glycerol esters	WITEPSOL H 15 [23]	-	-	33–36 [23]
Glyceryl behenate (SR)	Compritol^®^ 888 ATO [49,52,53,54] Compritol^®^ HD5 ATO [23]	2 [23,45] 5 [23]	- -	69–74 [45,52] 60–67 [23]
Glycerol monostearate (SR, Taste-Masking)	Geleol™ [52,55]	3 [52]	-	54–64 [52]
Glyceryl palmitostearate (SR)	Precirol ATO 5 [52,54,56]	2 [52]	-	50–60 [52] 52 [54]
Hydroxypropyl methylcellulose (SR)	HPMC K4M [54]	-	96 [48]	-
	HPMC K100M [12]	-	175 [12]	-
	AFFINISOL™ HPMC 100LV [57]	-	157 [57]	-
	AFFINISOL™ HPMC 15 LV [58]	-	97 [58]	-
	AFFINISOL™ HPMC 4 M [58]	-	96 [58]	-
	AFFINISOL™ HPMC 4M [57]	-	169 [57]	-
Hydroxypropyl methylcellulose acetate succinate (SR)	AQOAT^®^ LG [44]	-	122 [44]	-
Hydroxypropyl methylcellulose phthalate (SR)	HPMC Phthalate HP 55 [44]	-	145 [44]	-
Hydroxypropyl cellulose (IR, Compactability, SR)	Klucel^®^ MF [51,52]	-	120 [51]	-
	Klucel^®^ EF [51,59]	-	120 [48]	-
	Klucel^®^ ELF [49]	-	120 [49]	-
	Klucel^®^ EXF [49,60]	-	120 [49]	-
	Klucel^®^ HF [12]	-	130 [12]	-
	HPC-A [61]	-	<180 [61]	-
	HPC-S [61]	-	<180 [61]	-
Polymethacrylate copolymer (SR, Taste-Masking)	Eudragit^®^ RSPO [51]	-	64–66 [51]	-
	Eudragit^®^ EPO [44,58]	-	45–53 [44,58]	-
	Eudragit^®^ L100-55 [44]	-	110 [44]	-
Polyoxylglycerides (IR, SR, Solubilization)	Gelucire^®^ 50/13 [46]	11 [46]	-	50 [46]
	Gelucire^®^ 44/14 [62]	11 [46]	-	44 [46]
	Gelucire^®^ 43/01 [62]	1 [46]	-	43 [46]
	Gelucire^®^ 39/01 [62]	1 [46]	-	39 [46]
	Labrafil^®^ 2130CS [63]	9 [64]		52 [63]
Polyethylene glycol (IR, SR, Solubilization, Compactability)	PEG 400 [11,65]	-	-	53 [48]
	PEG 1500 [23]	-	-	44–48 [23]
	PEG 3350 [47,61]	-	-	62, 47–73 [47]
	PEG 4000 [11,65,66,67]	-	-	61.3, 45.2–67.2 [11]
	PEG 6000 [36]	-	-	55–60 [36]
	PEG 8000 [47]	-	-	63, 52–73 [47]
Polyethylene oxide (SR)	Sentry™ Polyox™ WSR 303 [68]	-	-	<75 [68]
	Polyox™ N10 NF [44]	-	-	65–70 [44]
	PEO 1M [54]	-	-	65–70 [54]
Poloxamer; Polyethylene oxide polypropylene oxide copolymer (Solubilization, Taste-Masking)	Kolliphor^®^ P188 [69]	>24 [23]	-	50.9 [69]
	Kolliphor^®^ P407 [37]	18–23 [23]	-	56 [37]
Polyvinyl pyrrolidone (SR)	Kollidon^®^ 12 PF [44]	-	72 [44]	-
	Kollidon^®^ 30 [44]	-	160 [44]	-
	Kollidon^®^ SR [68]	-	-	-
Vinylpyrrolidone-vinyl acetate copolymer (SR)	Kollidon^®^ VA64 [51]	-	108 [58]	-
Polyvinyl caprolactam–polyvinyl acetate–polyethylene glycol graft copolymer (SR, IR)	Soluplus [56,65,70]	- -	64 [58] 70 [70]	- -
Stearic acid (SR)	*Stearic Acid* [8,56,59,71,72]	15 [73]	-	69 [72,73]
Thermoplastic polyurethanes (SR)	Tecoflex™ EG72D [74]	-	-	55 [48]
	Tecophilic™ SP60D60 [74]	-	-	71 [48]

^1^ Sustained Release, ^2^ “-” indicates attribute not relevant, ^3^ Immediate Release.
